# Effective Degradation of Venlafaxine via Biochar Activated Persulfate: Kinetics, Transformation Products, and Toxicity Assessment

**DOI:** 10.3390/molecules30183720

**Published:** 2025-09-12

**Authors:** Alexandra A. Ioannidi, Eleni I. Panagopoulou, Konstantinos Kouvelis, Dimitrios Ladakis, Athanasia Petala, Marilena E. Dasenaki, Nikolaos S. Thomaidis, Dionissios Mantzavinos, Zacharias Frontistis, Olga S. Arvaniti

**Affiliations:** 1 Department of Chemical Engineering, University of Patras, Caratheodory 1, University Campus, 26504 Patras, Greece; alex.ioannidi@chemeng.upatras.gr (A.A.I.); koskouv@chemeng.upatras.gr (K.K.); mantzavinos@chemeng.upatras.gr (D.M.); 2 Laboratory of Analytical Chemistry, Department of Chemistry, National and Kapodistrian University of Athens, Panepistimioupolis Zografou, 15771 Athens, Greece; elenapanag@chem.uoa.gr (E.I.P.); ntho@chem.uoa.gr (N.S.T.); 3 Department of Agricultural Development, Agrofood and Management of Natural Resources, National and Kapodistrian University of Athens, 34400 Psachna, Greece; dladakis@agro.uoa.gr; 4 Department of Environment, Ionian University, 29100 Zakynthos, Greece; apetala@ionio.gr; 5 Laboratory of Food Chemistry, Department of Chemistry, National and Kapodistrian University of Athens, Panepistimiopolis Zographou, 15771 Athens, Greece; mdasenaki@chem.uoa.gr; 6 Department of Chemical Engineering, University of Western Macedonia, 50132 Kozani, Greece

**Keywords:** water treatment, antidepressants, venlafaxine, potato peel, biochar, catalysis, persulfate, transformation product, LC-HRMS, risk assessment

## Abstract

In this study, biochars (BC) from potato peel residuals were synthesized at 400, 600, and 800 °C, characterized, and evaluated for the persulfate-assisted oxidation of venlafaxine (VEN). BC pyrolyzed at 800 °C demonstrated the highest catalytic activity, resulting in the degradation of 750 μg/L of VEN in the presence of 500 mg/L persulfate in less than 90 min. Acidic conditions favored VEN destruction, while the apparent kinetic constant was reduced from 0.1136 at pH 3 to 0.0389 and 0.0352 min^−1^ for pH 7 and 9, respectively. Interestingly, the presence of inorganic ions such as bicarbonates and chlorides, as well as humic acid, only slightly reduced process efficiency. Scavenging tests and electron paramagnetic resonance spectroscopy indicate a mixed mechanism dominated by non-radical pathways, with minor radical contributions, mediated by oxygenated surface functionalities of the 800 °C biochar. Five transformation products were identified through LC-HRMS suspect and non-target approaches, and a potential degradation pathway was proposed. Most of the identified transformation products exhibited lower toxicity levels than the parent compound. Finally, life cycle analysis revealed that, despite its superior kinetics, the 800 °C biochar carries the largest environmental footprint, underscoring the need for integrated assessments that jointly optimize removal performance and environmental impacts.

## 1. Introduction

Venlafaxine (1-[2-(dimethyl amino)-1-(4-methoxy-phenyl)ethyl] cyclohexane hydrochloride, denoted hereafter as VEN, (brand name: Effexor XR, Lanvexin or Trevilor) is an antidepressant drug belonging to the general class of serotonin–norepinephrine reuptake inhibitors (SNRIs) [1,2]. VEN is officially approved to treat depression, anxiety, sleep, eating, and panic disorders by raising the concentrations of these compounds in the patient’s body and brain [3]. They are typically prescribed for long-term use and, therefore, synthesized in higher amounts than many other types of medications [3]. The average consumption of antidepressants in 18 European countries was 30.5 of the defined daily dose (DDD) per 1000 people per day in 2000, increasing to 75.3 DDD in 2020, a 147% rise, based on data from the Organisation for Economic Cooperation and Development (OECD). Iceland exhibited the highest consumption, with 153 DDD, followed by Portugal (131 DDD), the United Kingdom (108 DDD in 2017), Sweden (105 DDD), and Spain (87 DDD), while Latvia had the lowest with 20 DDD [4]. Greece, with 66 per 1000 people per day in 2020, recorded the highest increase (247%) since 2000 (19 DDD) [5]. Excretion of VEN and its metabolites occurs primarily through the renal route, ending up in sewage treatment plants (STPs), with only 5% of a venlafaxine dose appearing in the urine as an unchanged drug and 85% as active metabolites (N-desmethylvenlafaxine, O-desmethylvenlafaxine and N,O-didesmethylvenlafaxine). The apparent elimination half-life of VEN is 5 ± 2 h, and that of O-demethylvenlafaxine is 11 ± 2 h [6].

The presence of VEN and its metabolites in wastewater has been reported in numerous studies, with concentrations ranging from some ng/L up to 1 μg/L [7,8,9,10]. VEN, as expected, has also been found in surface water, tap water, and seawater [11,12,13]. For instance, in a Greek study by Kosma et al. [10], VEN was present at a concentration of 123 ng/L in influent wastewater and 66 ng/L in STP effluent, while its principal metabolite, O-desmethylvenlafaxine, was present at higher levels (281 in the influents and 85 ng/L in the effluents). Alygizakis et al. [9] studied the occurrence of 16 antidepressant drugs in seawater from the Eastern Mediterranean Sea. They found norvenlafaxine, one of the primary metabolites of VEN, in the range of (<0.01–2.0 ng/L) and with a detection frequency of 68.2%.

Persistent in the aquatic environment, VEN and some of its metabolites are considered among the most toxic substances to aquatic life, even at low concentration [14,15]. Elevated levels of VEN in the body are capable of damaging kidneys and liver and potentially causing epilepsy, which would negatively impact on the body and daily functions [16,17]. In general, there is a dearth of information regarding their ecotoxicological effects, and their transformation products (TPs) have not been described thoroughly in the literature.

Since treatment technologies used in conventional STPs have proven inadequate in removing pharmaceuticals like VEN [18,19,20], it is necessary to employ effective alternatives to protect water resources and the environment. Under this perspective, advanced oxidation processes (AOPs) seem to be gaining ground due to their simplicity, high oxidizing potential, and their green nature [21,22]. The oxidative power of AOPs is attributed to the reactive oxygen species, particularly hydroxyl radicals (${HO}^{•},E_{o}$ = 1.80–2.80 V, t_1/2_ < 1 μs), while other reactive species, such as sulfate radicals (${SO}_{4}^{•-}$, $E_{o}$ = 2.60–3.10 V, t_1/2_ = 30–40 μs) can also act either exclusively or as supplementary agents in different AOPs [23,24]. Sulfate radical generation derives from the activation of oxidants such as persulfates, peroxydisulfates, or peroxymonosulfates, using different methods, including heat (thermal, ultrasound, microwave), transition metal ions, UV radiation, and electrochemical processes [21,25].

Recently, biochar (BC), a prominent class of carbonaceous materials, has gained attention as an effective catalyst for persulfate activation in the degradation of organic pollutants, including pesticides and antibiotics [26,27]. Biochars are synthesized by pyrolyzing inexpensive agricultural waste such as sludge, forest residues, food waste, farming crop residues, and industrial organic waste—at moderate temperatures (e.g., 350–900 °C) under limited oxygen [26,27]. This process transforms biomass into a stable, carbon-rich material that resembles charcoal but has unique properties that make it extremely valuable in several applications. The primary advantages of BC for environmental applications are its large surface area, porous structure, thermal stability, and abundance of surface functional groups [27,28]. BC characteristics strongly depend on the synthesis conditions (such as pyrolysis temperature and time), as well as the waste materials used as carbon sources [27,28]. Employing materials that are by-products of other processes, such as biochar, is strongly encouraged in the context of sustainability and the circular economy for the integrated development of new systems and procedures [28,29].

Under this perspective, this study used potato peels as raw materials to prepare biochar and remove VEN from aqueous solutions. Potato (*Solanum tuberosum* L.) is a significant cash crop farmed worldwide, with millions consuming it in various forms regularly. The shift in consumer habits from fresh to processed foods, such as potato chips, snacks, puree, and French fries, has led to a rise in potato peel waste production by the processed food industry. Potato peels waste can vary between 15 and 40% of their initial weight, subject to the peeling method used, such as steam, lye, or abrasion. Furthermore, if potato peels are not handled properly, they decompose rapidly by bacteria or other biological forms, releasing an unpleasant odor and creating environmental pollution [30].

To date, few data are available on the degradation of VEN in water using AOPs such as UV/H_2_O_2_ [31], UV/Chlorine [32], TiO_2_-assisted photocatalysis [33], photo-Fenton [34], and electroperoxone [35]. Although reports on VEN abatement via persulfate exist (e.g., Fe^2+^/cysteine/PS [36], PTFE-supported rGO/PS [37], and CNT@Ni-Fe/Al_2_O_3_-cp-PVDF/PS [38]), many rely on transition metals and/or acidic pH and employ membrane-coupled configurations that raise cost and operational complexity. Here we advance a waste-derived biochar activator to favor simpler operation, wider pH applicability, and alignment with circular-economy principles.

In addition, most works studying the degradation of organic pollutants by AOPs have focused on kinetics and mechanistic aspects. On the other hand, an integrated assessment of the persulfate- and biochar-based AOPs implementing both the removal of pharmaceuticals and the environmental sustainability expressed as environmental footprint is missing from the relevant literature.

Life Cycle Assessment (LCA) methodology offers a comprehensive approach to environmental valuation, avoiding issues linked to impacts at multiple locations and periods in time [39]. Nevertheless, works assessing the ecological impact of treating pollutants with the PS-AOPs system using LCA are still scarce. To the best of our knowledge, this is the first time a comparative analysis of quantifiable environmental impacts has been conducted using the LCA of biochar and persulfate-AOPs for the elimination of VEN in aqueous matrices.

Thus, the present work aims to cover several identified gaps in the literature and to achieve the following goals: (i) Synthesize biochar from potato peels at different pyrolysis temperatures and characterize it using the Brunauer–Emmett–Teller (BET) method, X-ray diffraction (XRD), scanning electron microscopy (SEM), and Fourier-transform infrared (FTIR) spectroscopy. (ii) Assess the effect of different parameters such as biochar dose, pH, SPS concentration, initial VEN concentration, organic matter, inorganic ions, and water matrix on VEN degradation. (iii) Investigate the degradation mechanism, transformation products (TPs), and propose a primary pathway mechanism of VEN degradation in the PS–biochar-AOPs system. (iv) Explore the toxicity of VEN and its degradation products; (v) Use LCA to determine the environmental impacts.

## 2. Results

### 2.1. Physicochemical Characterization

**Figure 1:**
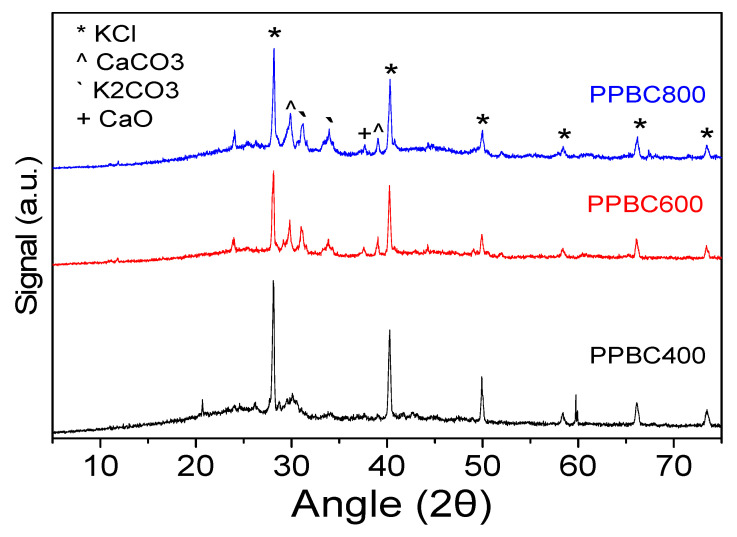
XRD patterns of PPBC samples.

The crystallinity and phase composition of the potato peel biochar (PPBC) samples were investigated using XRD, as shown in Figure 1. All three samples exhibit distinct features associated with increasing thermal treatment, reflecting the progressive transformation of the organic matrix and the formation of inorganic crystalline residues. The XRD pattern of PPBC400 is characterized by a broad, low-intensity peak located at 2θ ~22–25°, indicative of amorphous carbonaceous structures. In addition, the diffraction peaks at 28.2°, 40.3°, 49.9°, 66.24°, and 73.61° are indexed to KCl (JCPDS No 411476). The increase in pyrolysis temperature to 600 °C (PPBC600) led to the formation of sharp diffraction peaks, particularly at 2θ 28–32°, 34°, 47°, and 56°, revealing the formation of additional inorganic crystalline phases. These peaks can be attributed to potassium and calcium-based mineral phases, such as KCl, K_2_CO_3_, CaO and CaCO_3_ [30,40]. Specific peaks located at 29.9° and 39° can be attributed to CaCO_3_ (JCPDS No 3612), while the diffraction peak at 37.7° matches with the CaO pattern (JCPDS No 11160). The diffraction peak located at 34° can be indexed to K_2_CO_3_ (JCPDS No 491093). The presence of these crystalline residues is often associated with the thermal decomposition of plant-derived minerals and seems to play an important role in the catalytic efficiency of biochar. At the highest pyrolysis temperature, (PPBC800), the intensity and sharpness of the diffraction peaks increase further, suggesting enhanced crystallinity of the inorganic residues. The peak positions remain largely consistent with those observed at 600 °C, implying stabilization of the crystalline phase composition. In addition, the primary crystalline size of all samples was estimated according to the Scherrer equation, and the results are shown in Table 1. It is observed that PPBC400 and PPBC600 are characterized by the same primary crystallite size equal to ~41 Å, while thermal treatment at 800 °C leads to a slight decrease in crystallite diameter (~36 Å).

**Figure 2:**
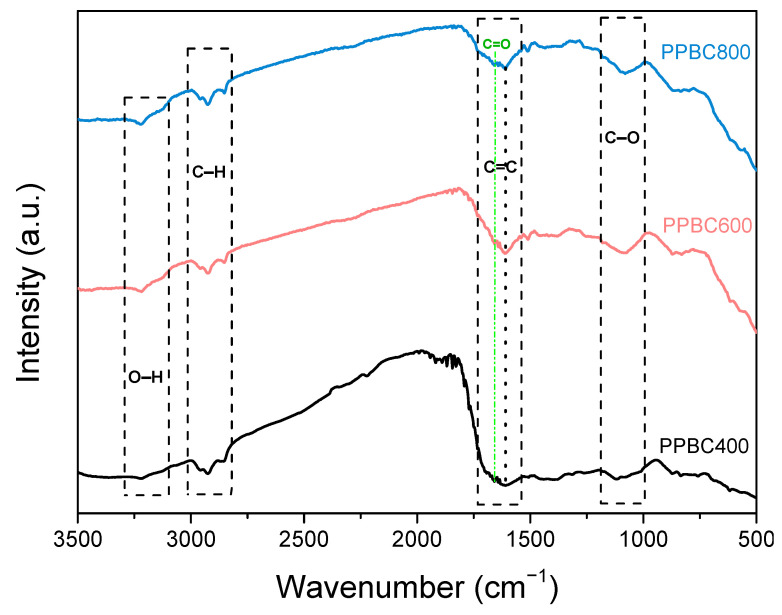
FTIR reflectance spectra of the potato peel-derived biochars.

The FTIR spectra of the synthesized catalysts (Figure 2) display several characteristic absorption bands. A narrow band appears at around 3200 cm^−1^, corresponding to O–H stretching vibration, typically associated with adsorbed moisture. In the region between 2850 and 2950 cm^−1^ a broader band is observed, attributed to the symmetric and asymmetric vibrations of C–H bonds present in aliphatic groups (e.g., –CH_2_ and–CH_3_). The presence of these features indicates the retention of cellulosic and hemicellulosic structures in the biochar. In addition, a distinct band is observed at approximately 1600 cm^−1^, attributed to the stretching vibrations of the C=C bond found in aromatic groups. Finally, several weaker bands appear between 1100–120 cm^−1^ corresponding to C–O stretching vibrations, commonly associated with alcohols, ethers and esters [30]. Additionally, as the pyrolyzed temperature increased from 400 to 800 °C a weak narrow band at around 1700 cm^−1^ starts to become visible, corresponding to C=O stretching vibration.

**Figure 3:**
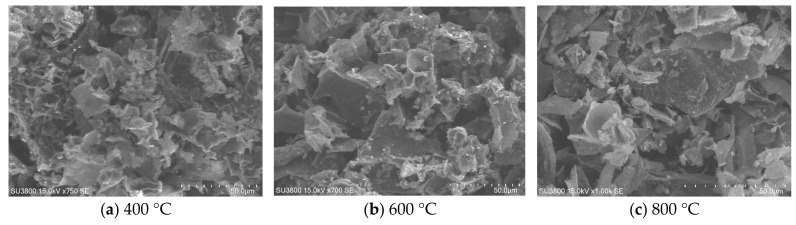
SEM images of PPBC calcined at (**a**) 400 °C, (**b**) 600 °C, and (**c**) 800 °C.

The surface morphology of potato peel-derived biochar samples calcined at 400 °C, 600 °C, and 800 °C and was investigated using SEM and representative images, as shown in Figure 3. It is observed that PPBC400 is characterized by a relatively smooth and dense surface, with a small number of observable pores or cracks, suggesting limited volatilization of organic components. The increase in calcination temperature to 600 °C led to higher surface heterogeneity, with pore formation and roughness indicative of progressive thermal decomposition of volatile matter. In addition, PPBC800 exhibited a markedly fragmented and porous structure. These morphological changes reflect the increasing carbonization and degradation of organic matter with temperature, potentially enhancing radical generation and degradation efficiency in carbon-based [30,40,41].

The specific surface area of the potato peel-derived biochar exhibited a relatively low SSA of 1.7 m^2^/g at 400 °C, in line with former studies on plant derived biochar [42,43]. Increasing the pyrolysis temperature to 600 °C and 800 °C did not lead to a substantial change in SSA, with the PPBC800 sample having SSA equal to 2.5 m^2^/g. This observation may be attributed to the intrinsic composition of potato peels, which can result in the formation of a more compact and less porous carbon matrix during pyrolysis.

### 2.2. Catalytic Results

#### 2.2.1. Effect of Pyrolysis Temperature on the Catalytic Activity of PPBC

**Figure 4:**
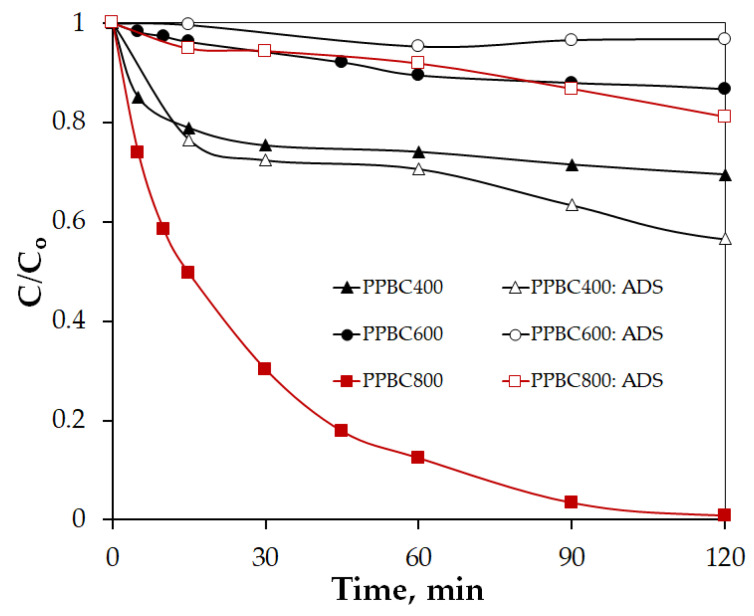
Effect of pyrolysis temperature on catalytic and adsorption performance of PPBC for the removal of VEN. Experimental conditions: [PPBC400] = [PPBC600] = [PPBC800] =500 mg/L, [VEN] = 0.75 mg/L, [SPS] = 500 mg/L, pH = 7 in UPW.

To assess the impact of pyrolysis temperature on biochar’s catalytic and adsorption performance for the removal of VEN, three temperatures ranging from 400 °C to 800 °C were examined. Figure 4 and Table 2 depict the results, which reveal that adsorption has a minimal contribution. Only PPBC400 achieved 44% VEN removal after 120 min through adsorption. The remaining biochar samples exhibited adsorption efficiencies ranging from 3% (PPBC600) to 19% (PPBC800). This behavior may be due to the amorphous carbonaceous structure of PPBC400, which retains polar functional groups that enhance the adsorption of polar to moderately polar compounds, such as venlafaxine hydrochloride (logP = 0.43). As the pyrolysis temperature increases, these polar groups (e.g., C–O, –OH) are reduced, while graphite-like carbon structures and overall hydrophobicity increase. This shift leads to lower adsorption efficiency for moderately polar molecules [44]. It is worth noting that the observed results are in agreement with the physicochemical characterization of PPBC400, PPBC600, and PPBC800 as presented in Section 2.1.

With respect to the catalytic performance of PPBCs, both PPBC400 and PPBC600 exhibited negligible pollutant degradation under the tested condition. Notably, PPBC400 not only was unable to activate persulfate, but also showed a slightly lower VEN removal compared to adsorption alone. This effect may be attributed to the adsorption of excess SPS molecules onto the biochar surface, which likely hindered the availability of active sites for VEN adsorption. In contrast, at 800 °C, the catalytic activity of the biochar led to complete VEN decomposition within 120 min. The high catalytic efficiency of PPBC800 may be due to a slight increase in SSA, smaller crystallite size, and higher graphitic carbon content, which probably facilitated electron-mediated reactions [45]. Based on the above findings, PPBC800 was selected for further investigation.

#### 2.2.2. Factors Affecting the Performance of the PPBC/Persulfate System

**Figure 5:**
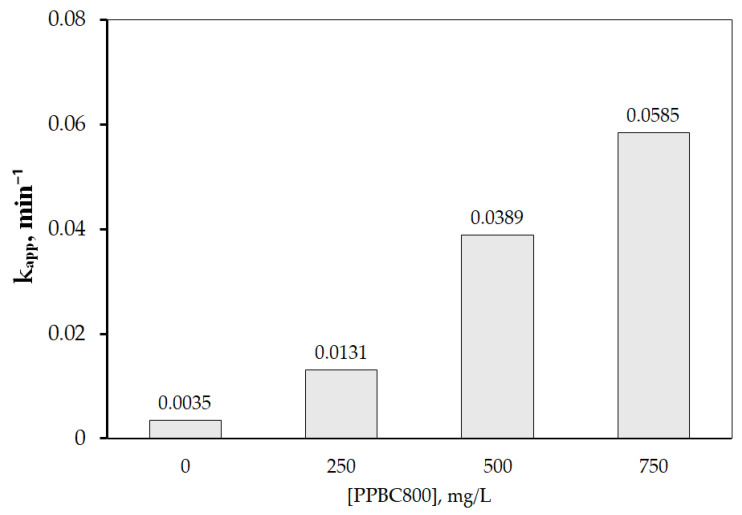
Effect of initial concentration of PPBC800 on VEN degradation in UPW. Experimental conditions: [SPS] = 500 mg/L and [VEN] = 0.75 mg/L at pH 7.

In addition, the effects of PPBC and SPS dosage on VEN degradation were explored, and the degradation kinetics were analyzed using a pseudo-first-order reaction model. Generally, a higher biochar dosage enhances efficiency by providing more active sites for persulfate activation. As shown in Figure 5, a positive correlation was observed between catalyst dosage (ranging from 250 mg/L to 750 mg/L) and degradation rate. At the highest dosage of 750 mg/L, complete degradation of VEN was achieved within 90 min.

VEN degradation followed a pseudo-first-order kinetics at all tested biochar dosages (R^2^ > 0.99). As the biochar dosage increased from 250 to 750 mg/L, the apparent (observed) rate constant (k_app_) rose from 0.0131 to 0.0585 min^−1^. Approximately 35% of VEN was removed within 120 min by the sodium persulfate alone, indicating that persulfate is ineffective for VEN degradation without a catalyst due to the relatively low redox potential of persulfate compared to reactive species and radicals produced in the presence of biochar. These results are consistent with previous studies, which have demonstrated that higher catalyst dosages can accelerate the degradation rate of organic pollutants [46].

**Figure 6:**
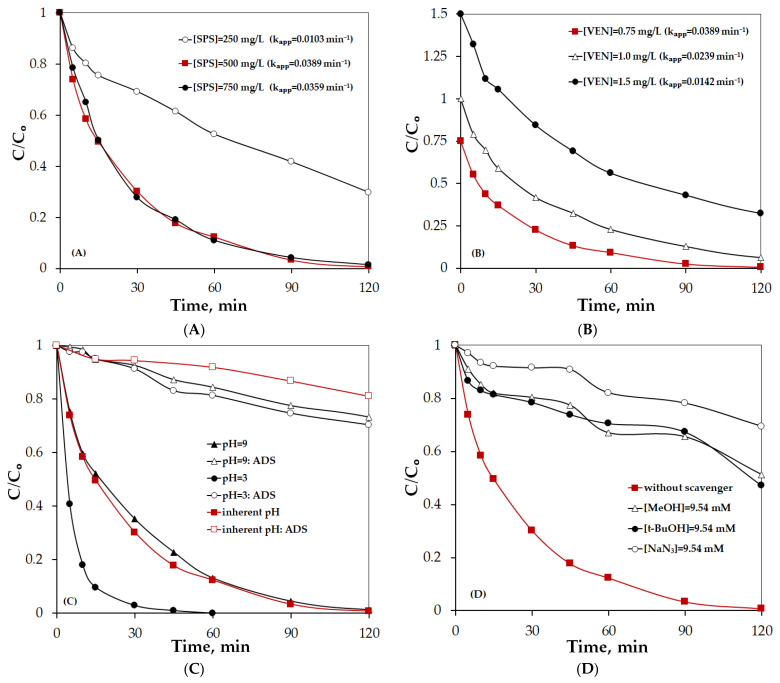
(**A**) Effect of initial concentration of SPS on the degradation of 0.75 mg/L VEN with 500 mg/L PPBC800 at pH = 7 in UPW. (**B**) Effect of initial concentration of VEN on its degradation with 500 mg/L PPBC800 and 500 mg/L SPS at pH = 7 in UPW (numbers in brackets show apparent rate constants). (**C**) Effect of initial pH solution on the degradation and adsorption of 0.75 mg/L VEN. Experimental Conditions: [PPBC800] = 500 mg/L, and [SPS] = 500 mg/L in UPW. (**D**) Effect of scavengers on 0.75 mg/L VEN degradation with 500 mg/L PPBC800 and 500 mg/L SPS in UPW.

Furthermore, another strategy to enhance process efficiency is the increase in the concentration of the oxidant and, consequently, the production of more reactive species. As shown in Figure 6, an increase in SPS concentration from 250 mg/L to 500 mg/L led to an enhancement in VEN degradation efficiency from 86% to 100% after 120 min of reaction time, resulting in a 2.5-fold increase in the k_app_ values, with the respective k_app_ being 0.0163 min^−1^ at 250 mg/L SPS and 0.0389 min^−1^ at 500 mg/L SPS (Figure 6A). However, once the oxidant concentration surpasses 500 mg/L, the degradation performance remains nearly unchanged. This phenomenon is likely due to the self-quenching of reactive species. Therefore, bearing the associated cost and the production of sulfate ions as by-products in mind, an SPS concentration of 500 mg/L was selected to examine the subsequent operating parameters in UPW.

The role of the initial concentration of VEN on its degradation was investigated by varying VEN concentration between 0.75 and 1.5 mg/L, while keeping the concentrations of biochar and SPS constant at 500 mg/L. Figure 6B shows that the PPBC800/SPS process can degrade 0.75 mg/L VEN in 120 min. From an engineering point of view, as the VEN concentration increases, the quantity of VEN degraded in the system also increased, achieving 0.935 mg/L and 1.177 mg/L VEN removal at 120 min for the initial concentration of VEN 1 mg/L and 1.5 mg/L, respectively. In terms of kinetics, a reduction in k_app_ values was observed from 0.0389 min^−1^ at 0.75 mg/L to 0.0142 min^−1^ at 1.5 mg/L VEN, proving that VEN decomposition follows a pseudo-first-order model rather than a true first-order one, where the kinetic constant is independent of the pollutant’s initial concentration. This behavior is quite common in AOPs and is mainly attributed to limitations in mass transfer or reactive species formation [46,47].

The pH is a significant operating parameter and plays a complex role in activated persulfate systems since it affects both the types of reactive species formed and their redox potential, as well as the surface charge of biochar and the charge of the target compound [48,49]. The effect of solution pH (3–9) on the degradation of 0.75 mg/L VEN was investigated, and the results are presented in Figure 6C and Table 3. As observed, the most efficient degradation of VEN occurred at pH 3, while the system demonstrated high pH tolerance, maintaining steady degradation efficiency across pH values between 7 and 9. The corresponding k_app_ values are as follows: 0.1136 > 0.0389~0.0352 min^−1^ at pH values of 3, 7, and 9, respectively. Combining the adsorption and oxidation performance (Figure 6C) suggests that the adsorption capacity of the PPBC800/SPS system had practically a negligible role in the process efficiency. Similar results were reported by Wei et al. [49], who studied the degradation of sulfamethoxazole by N-doped iron-based carbon-activated peroxymonosulfate at several pH solutions.

The superior VEN degradation at pH 3 arises from the pH-regulated activation of persulfate on the carbon surface. Across diverse carbons, kinetics of persulfate-based advanced oxidation processes typically follow pH ≈ 4 > 7 > 9, since increasing pH hinders PDS–carbon interactions and suppresses the formation of surface-bound oxidants (radical and non-radical). This trend is consistent with our system, in which singlet oxygen plays a dominant role, as we will discuss later. By contrast, alkaline pH promotes the conversion of sulfate radicals to hydroxyl radicals and attenuates the interfacial electron-transfer regime, resulting in slower VEN decomposition despite similar VEN speciation across pH 3–7 [50].

To elucidate the possible mechanism of the PPBC800/persulfate system, quenching tests with methanol, tert-butyl alcohol, and sodium azide were conducted. Specifically, 9.54 mM of MeOH, t-BuOH and NaN_3_ were used to quench ${SO}_{4}^{•-}$, ${HO}^{•}$, and ^1^O_2_, respectively. The concentration of scavengers was excessive compared to the SPS concentration (Table 4). As shown in Figure 6D, at pH 7, the inhibition of the VEN degradation rate in the presence of t-BuOH and MeOH was nearly identical, while the addition of NaN_3_ led to an even greater reduction in VEN oxidation compared to the performance without any scavenger.

**Figure 7:**
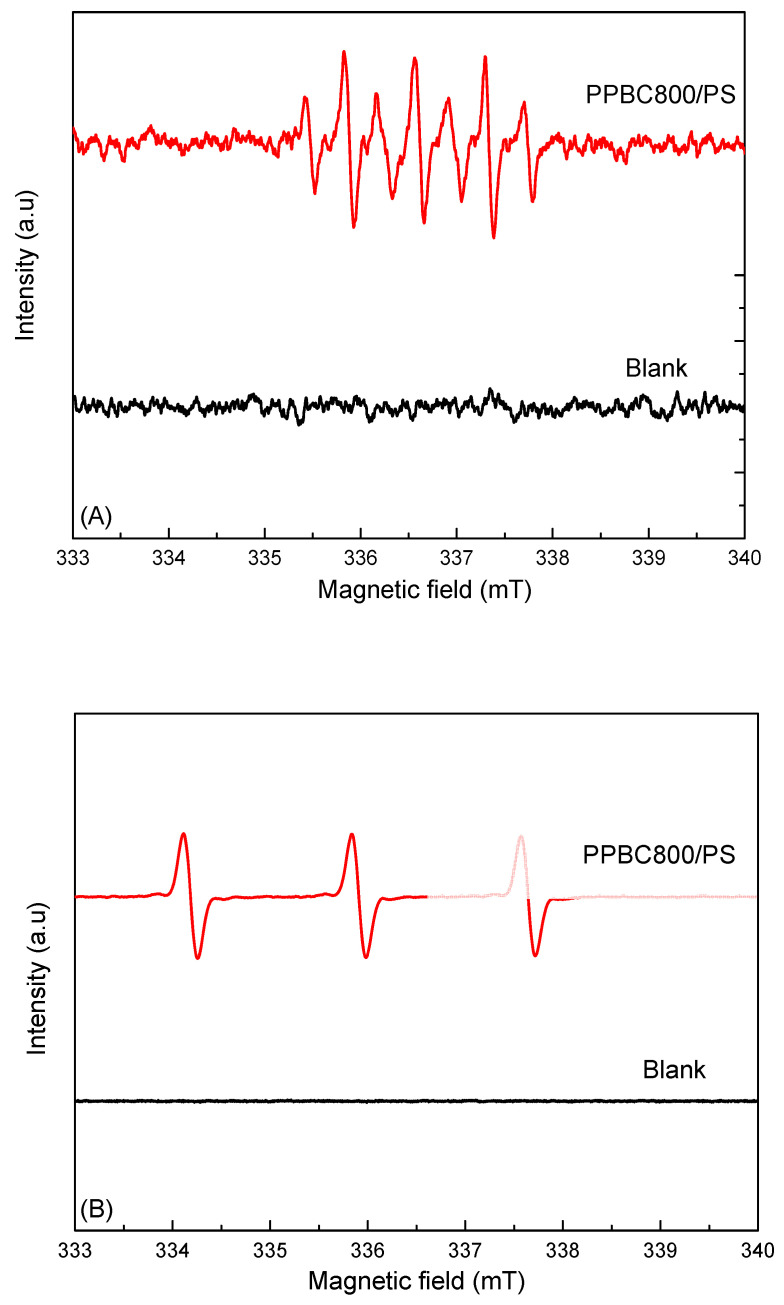
EPR spectra obtained with different spin traps: (**A**) DMPO and (**B**) TEMP.

To further elucidate the reaction mechanism, additional EPR measurements were performed using DMPO (radical spin trap) and TEMP (singlet-oxygen probe) and representative spectra are shown in Figure 7. The DMPO spectra exhibited the broad, seven-line DMPO-X pattern rather than the characteristic DMPO–${SO}_{4}^{•-}$ or DMPO–^•^OH adducts typically observed in radical-dominated persulfate systems. Given the absence of these adducts and the prevalence of the DMPO–X signature (arising from secondary oxidation of DMPO), radical pathways do not dominate under our conditions. Oxidation of TEMP to TEMPO was clearly detected, providing direct evidence for the generation of singlet oxygen in the PPBC800/persulfate system. Taken together, the EPR data, in concert with the scavenging results, indicate a mixed radical/non-radical pathway, with any ${SO}_{4}^{•-}$/^•^OH contribution likely minor or short-lived and below our detection limit, while non-radical oxidation (via ^1^O_2_ and surface-mediated electron transfer) prevails.

This interpretation aligns with the functional group analysis and supports the role of oxygenated surface sites on the 800 °C biochar in driving VEN degradation. As already discussed, FTIR shows the emergence of C=O groups (≈1700 cm^−1^) together with retained aromatic C=C (~1600 cm^−1^) and C–O bands (≈1100–1200 cm^−1^) in PPBC800, and SEM indicates a more fragmented/porous morphology relative to lower-temperature biochars. While these techniques cannot directly resolve electronic defect states, the combination of oxygenated functionalities and conjugated carbon domains can facilitate surface-mediated electron transfer to persulfate, enabling a non-radical route that yields ^1^O_2_ alongside limited ${SO}_{4}^{•-}$ and ^•^OH chemistry [47,50,51,52].

#### 2.2.3. Impact of Natural Water Constituents on the PPBC800/Persulfate System

A complex array of inorganic anions, cations, and natural organic matter (NOM) is present in natural waters that can inhibit or promote process efficiency, mainly due to the scavenging or alteration of the produced reactive species [53,54,55].

**Figure 8:**
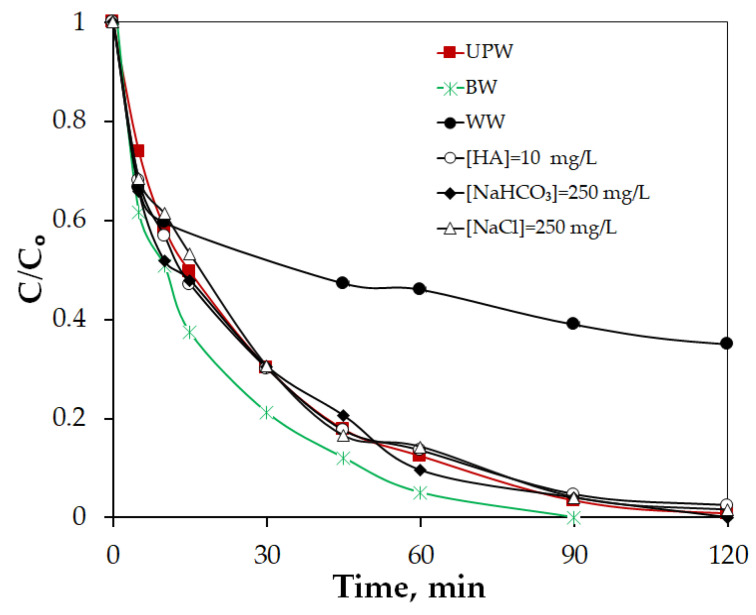
Effect of water matrix on the degradation of 0.75 mg/L VEN with 500 mg/L PPBC800 and 500 mg/L SPS.

In this study, the effect of inorganic anions on VEN degradation was evaluated by adding ${HCO}_{3}^{-}$ and ${Cl}^{-}$ at fixed concentrations of 250 mg/L. As shown in Figure 8, the addition of ${HCO}_{3}^{-}$ resulted in negligible changes in VEN removal. Although ${HCO}_{3}^{-}$ can act as a radical scavenger by reacting with ${HO}^{•}$, and ${SO}_{4}^{•-}$ to form carbonate radicals (${HCO}_{3}^{•}$/${CO}_{3}^{•-}$), which typically have lower reactivity than ${HO}^{•}$ and ${SO}_{4}^{•-}$, their longer lifetime and higher steady-state concentration in aqueous solution are sometimes considered significant contributors in radical assisted processes [56,57,58]. However, in the present case, such effects were not substantial. Similarly, ${Cl}^{-}$ addition also did not affect VEN degradation (Figure 8). While according to several studies ${Cl}^{-}$ can be oxidized by the primary radicals to form reactive chlorine species (e.g., ${Cl}^{•}$, ${Cl}_{2}^{•-}$, and ${ClOH}^{•-}$) and free chlorine (e.g., Cl_2_, HOCl, and OCl^−^), these species are generally weaker oxidants and may lead to the formation of chlorinated byproducts [59,60,61]. In this study, their formation did not significantly influence VEN removal, suggesting that any scavenging of ${SO}_{4}^{•-}$, and/or ${HO}^{•}$ by ${Cl}^{-}$ was either minimal or effectively balanced by secondary oxidation pathways, resulting in no observable impact on degradation performance.

In addition, 10 mg/L humic acid (HA) was added as a representative of aquatic NOM to examine its effect on VEN degradation. It is evident in Figure 8 that the presence of HA did not affect the degradation of VEN, and this could be at least partially attributed to the observation that the non-radical pathway plays an important role in VEN elimination in the PPBC800/SPS system, as already discussed in Section 2.2.2.

Finally, VEN degradation effectiveness in real water matrices was investigated to assess the technological feasibility of the proposed system for decomposing VEN in the aquatic environment. Therefore, VEN degradation was tested at 0.75 mg/L, at least two orders of magnitude greater than its normal concentration range across most contaminated wastewater, while the water samples’ pHs were approximately 8. By principle, non-target elements compete with pollutants for oxidizing species, resulting usually in reduced pollutant degradation. Notably, as shown in Figure 8, the BW matrix accelerated VEN degradation, whereas the WW matrix moderately inhibited it. For example, 100% of VEN was degraded in UPW after 120 min in the PPBC800/SPS system, compared to 65% in WW, respectively. Since the effect of pH is excluded, the slightly lower rate in WW likely reflects the various inorganic and organic constituents present in wastewater (measured as 7 mg/L of TOC and 21 mg/L of COD) that can reduce efficiency.

Comparison with representative recent studies (Table S1, Supplementary Information) indicates that, although faster removal is often reported for energy-assisted or metal-containing persulfate systems, these typically require narrow pH control or more complex configurations. In contrast, the present metal-free process, based on the valorization of waste biomass to biochar, maintains activity at near-neutral pH with only minor inhibition by common matrix constituents; together with the absence of external energy inputs and added metals, this aligns with circular-economy principles and offers a low-tech, green option with applicability in realistic water treatment.

### 2.3. Identification of Transformation Products

**Figure 9:**
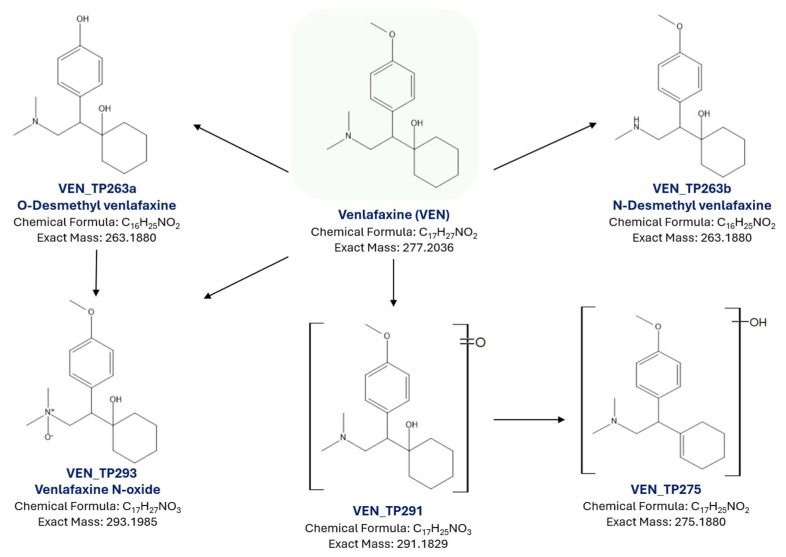
Proposed structures and transformation pathways of VEN degradation.

The screening of the samples reveals the presence of five transformation products of venlafaxine with reversed-phase high-resolution liquid chromatography-mass spectrometry (LC-HRMS). The TPs were detected through an in-house suspect screening workflow, whereas no additional transformation product was detected through non-target screening. Additionally, all the TPs were detected utilizing the complementary chromatographic technique, HILIC, which enhanced identification confidence. The identification of each tentative TP was supported by different analytical evidence, and different confidence levels for the proposed structures were reached. TPs were detected with a higher intensity as [M + H]^+^ ion utilizing positive ionization analysis, while no additional TPs were found in negative ionization mode. An overview of the detected TPs, along with relevant information, is reported in Table 5. The proposed structures of the detected TPs and the potential formation pathway are illustrated in Figure 9. Furthermore, the MS/MS spectra of these identified TPs acquired with data-dependent acquisition (DDA) mode are presented in Figure S1.

The extracted ion chromatogram of the [M + H]^+^ ion with *m*/*z* 264.1958 ([C_16_H_26_NO_2_]^+^) reveals the presence of two distinct chromatographic peaks. The potential transformation products VEN_TP263a and VEN_TP263b were eluted at 4.51 and 5.53 min, respectively, using the RPLC system. Both compounds appear to result from the demethylation of venlafaxine. Based on the molecular structure, demethylation may occur at two different sites, either on the methoxy group (O-demethylation) or on the N-methyl group (N-demethylation) [31,62,63].

The identification of these transformation products indicates a thorough evaluation of the MS/MS spectra. The MS/MS spectra of VEN_TP263b exhibited the characteristic fragment ions with *m*/*z* 121.0648, [C_8_H_9_O]^+^ and *m*/*z* 147.0804 [C_10_H_11_O]^+^, which follow the fragmentation profile of the parent compound VEN and the existing literature [31,62,63], enhancing the identification confidence. These fragments indicated the presence of an aromatic moiety and suggest that demethylation occurred in the N-methyl group, resulting in the formation of N-desmethyl venlafaxine. This is a characteristic transformation product of VEN that has previously been reported in the literature [31,33,63,64,65].

In contrast, the evaluation of the MS/MS spectrum of VEN_TP263a reveals the fragment with *m*/*z* 107.0886, [C_8_H_11_]^+^ and *m*/*z* 133.0886 [C_9_H_11_N]^+^, indicating that this TP follows a different fragmentation pathway [62,63]. These fragments suggest that demethylation occurred at the methoxy group on the aromatic ring, whereas the methoxy group (–OCH_3_) on the aromatic ring converted into a hydroxylic group. Both transformation products are commonly detected as metabolites of venlafaxine [31,33,63,64,65,66,67]. Therefore, analytical standards of O-desmethyl venlafaxine and N-desmethyl venlafaxine were purchased, analyzed, and used for their identification in the samples. The alignment of retention times and the matching MS/MS fragmentation patterns among the detected TPs, VEN_TP263a,b, and the analytical standards confirmed their structure elucidation. Furthermore, confirmatory data from databases and literature sources further substantiated the confirmation of these TPs, achieving identification level 1 [68,69]. Therefore, VEN_TP263a was confirmed as O-desmethyl venlafaxine and VEN_TP263b as N-desmethyl venlafaxine.

Moreover, VEN_TP293, with a molecular mass of C_17_H_28_NO_3_^+^ and an *m*/*z* 294.2063, was also identified through suspect screening and eluted at 6.27 min. The mass of the VEN_TP293 molecule was observed to be 16 Da higher than that of VEN (278.2124 *m*/*z*), which corresponds to the mass of an oxygen atom, indicating that oxidation was performed. The evaluation of the MS/MS spectrum reveals the main fragment ions with *m*/*z* 121.0648, [C_8_H_9_O]^+^ and *m*/*z* 215.1430 [C_15_H_19_O]^+^, which are consistent with the existing literature [33,36,63]. Notably, oxidation appears to have taken place at the nitrogen atom of the amine group, as evidenced by the fragment ion at *m*/*z* 178.1226 [C_15_H_19_O]^+^, which is characteristic of an N-oxide structure. Since the analytical standard was available, this TP was confirmed as N-oxide venlafaxine.

Regarding VEN_TP291, it was detected as *m*/*z* 292.1912 utilizing the RPLC system and eluted at 7.85 min. This compound, with a molecular mass of C_17_H_25_NO_3_, has previously been reported as a transformation product of venlafaxine [33,63,66]. The characteristic fragments with *m*/*z* 121.0648 and *m*/*z* 215.1430 [C_15_H_19_O]^+^ were predominant. These fragments have been previously reported and detected in MS/MS spectra of the parent compound and the aforementioned TPs. The formation of VEN_TP291 is attributed to an oxidation reaction, likely introducing an additional oxygen atom.

Furthermore, the extracted ion chromatogram of the *m*/*z* 276.1958 revealed a chromatographic peak at 4.7 min, which corresponds to VEN_TP275 with a molecular mass of C_17_H_25_NO_2_, and has been reported in the literature [33]. Its MS/MS spectrum showed a characteristic fragment *m*/*z* 147.0676 [C_9_H_9_NO]^+^. Along with that, a more abundant fragment ion with *m*/*z* 162.0913 [C_10_H_12_NO]^+^ was detected; however, it did not provide helpful information for the structure of the compound. Based on the observed *m*/*z* and the fragmentation pattern, VEN_TP275 likely results from a combination of dehydration followed by hydroxylation or potentially dihydroxylation of VEN-TP294, suggesting that an oxygen atom had been introduced into the parent compound together with a subsequent H_2_O loss.

**Figure 10:**
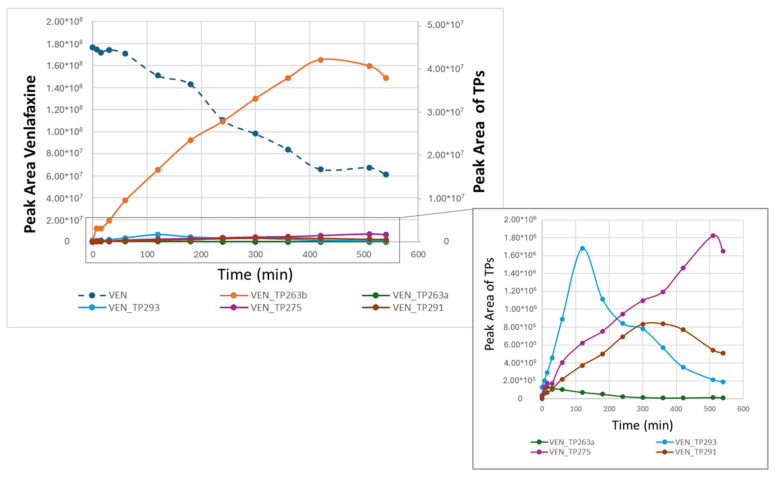
Time-trend profiles of venlafaxine and its detected transformation products through RPLC-ESI(+)-TIMS-QToF analysis.

In addition, the time-trend profiles of venlafaxine and its TPs degradation obtained through RPLC-ESI(+)-TIMS-QToF analysis are presented in Figure 10. These profiles reveal distinct behaviors among the various TPs formed during the degradation process. The most abundant transformation product was VEN_TP263b (N-desmethyl venlafaxine), which exhibited a continuous and steady increase in abundance with time, reaching a maximum at the end of the experiment (510 min). This pattern suggests that VEN_TP263b is a relatively stable metabolite that accumulates as venlafaxine is progressively degraded. Similarly, VEN_TP275 followed the same trend, with a gradual increase in abundance over the experiment. In contrast, VEN_TP293 revealed a different time-trend profile, reaching its maximum abundance within the first 120 min of the experiment, followed by a rapid decrease. This pattern suggests that VEN_TP293 may subsequently be transformed or further degraded into other transformation products. Also, VEN_TP291 reached the maximum levels in the middle of the experiment, indicating that it may arise as a secondary transformation product. Finally, VEN_TP263a was detected at low abundance initially but increased sharply within the first 15 min. This observation supports the hypothesis that VEN_TP263a could serve as a precursor or intermediate in the sequential transformation process leading to other, more abundant TPs.

### 2.4. Toxicity Assessment of Transformation Products

The evaluation of the ECOSAR model reveals that all the TPs exhibit lower toxicity levels than the parent compound VEN, which follows the reported data in the literature [67].

Among the TPs, VEN_TP263b and VEN_TP275 demonstrated the highest toxicity across all tested aquatic organisms compared to the rest of the detected TPs, as detailed in Table 6. Acute toxicity values for most of the TPs and the chronic toxicity values of VEN_TP291 were classified as “harmful” towards fish, daphnia and green algae.

### 2.5. Life Cycle Assessment

**Figure 11:**
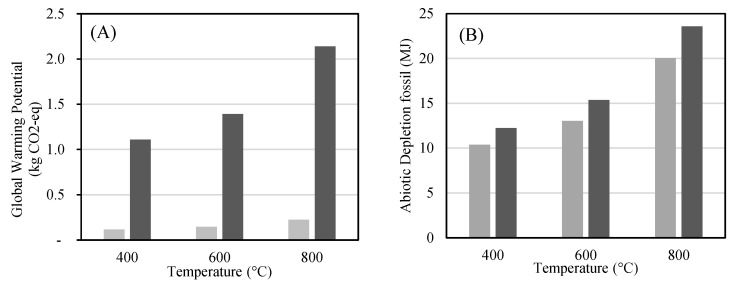
(**A**) Global Warming Potential (GWP, kg CO_2_-eq) and (**B**) Abiotic Depletion Potential–fossil (ADP-fossil, MJ) per kg of biochar produced at three pyrolysis temperatures (400 °C, 600 °C, and 800 °C), using either ∎ natural gas or electricity from the ∎ European grid as the energy source.

The Life Cycle Assessment results for the production of 1 kg of biochar from potato peels under three different pyrolysis temperatures (400 °C, 600 °C, and 800 °C) are presented in Figure 11, for the indicators Abiotic Depletion Potential–fossil fuels (ADP-fossil) and Global Warming Potential (GWP), respectively. Each scenario was evaluated for two energy supply systems: natural gas and electricity from EU grid mix.

Regarding climate change impacts, Figure 11A shows that GWP values are drastically higher for electricity-based systems, especially at 800 °C, where emissions exceed 2.1 kg CO_2_-eq/kg biochar, compared to only 0.23 kg CO_2_-eq in the natural gas scenario. In contrast, the GWP for natural gas remains below 0.3 kg CO_2_-eq across all temperatures, underlining its comparatively lower carbon footprint in this specific setup. As illustrated in Figure 11B, the ADP-fossil values increase significantly with higher pyrolysis temperatures, reflecting the higher energy requirements of the process. For instance, at 400 °C, fossil energy demand ranges from 10.3 MJ (NG) to 12.5 MJ (electricity) per kg of biochar, while at 800 °C it reaches 20.0 MJ (NG) and 23.3 MJ (electricity). This trend confirms the expected intensification of thermal input at elevated temperatures. Electricity-based systems consistently present higher ADP values compared to natural gas due to the fossil-based nature of the European grid mix. Despite the superior environmental performance of low-temperature pyrolysis in both ADP and GWP terms, the choice of operational conditions must be balanced with biochar performance criteria. As indicated by our experimental data, the biochar produced at 400 °C and 600 °C showed limited adsorption capacity and contaminant degradation potential, whereas the material obtained at 800 °C exhibited substantially higher surface area, porosity, and pollutant retention, making it more suitable for environmental applications at least in the conditions in question.

Therefore, while higher pyrolysis temperatures are associated with increased environmental burdens during production, they may offer enhanced functional performance in downstream use, especially in environmental remediation. This trade-off must be evaluated through a broader consequential LCA framework in future studies.

## 3. Materials and Methods

### 3.1. Chemical Reagents

Venlafaxine (VEN, C_17_H_27_NO_2_HCl, 98%, CAS: 93413-69-5), sodium persulfate (SPS, Na_2_S_2_O_8_, 99.9%, CAS: 7775-27-1), sodium bicarbonate (NaHCO_3_, ≥99%, CAS: 144-55-8), sodium chloride (NaCl, ≥99.8%, CAS: 7647-14-5), LC-grade humic acid (HA) (CAS: 68131-04-4), sodium hydroxide (NaOH, ≥96%, CAS:1310-73-2), sulfuric acid (H_2_SO_4_, 97%, CAS: 7664-93-9) phosphoric acid (H_3_PO_4_, 85%, CAS: 7664-38-2), sodium azide (NaN_3_, CAS: 26628-22-8), tert-butyl alcohol (t-BuOH, C_4_H_10_O, 99%, CAS: 75-65-0), methanol (MeOH, CH_3_OH, 99.9%, CAS: 67-56-1), and acetonitrile (ACN, CH_3_CN, 99.5%, CAS: 75-05-8), were all sourced from Sigma-Aldrich (St. Louis, MO, USA). Ammonium formate (≥99.0%, CAS: 540-69-2), ammonium acetate (99% CAS: 631-61-8), and formic acid (99%, CAS: 64-18-6) were all purchased from Fluka (Buchs, Switzerland).

The analytical standards of the transformation products, venlafaxine-N-oxide (CAS: 1094598-37-4), O-desmethyl venlafaxine (CAS: 93413-62-8), and N-desmethyl venlafaxine (CAS: 149289-30-5) were purchased from LGC (Mercatorstrass, Germany).

The Milli-Q water purification system (Millipore Direct-Q UV, Bedford, MA, USA) produced ultrapure water with a conductivity of 0.056 μS/cm and pH of 6. Wastewater samples (WW) were collected from the secondary treatment of STP situated within the University of Patras campus, and commercially bottled water (BW) was also utilized. The physicochemical characteristics of BW and WW can be found in the work of Ioannidi et al. [69].

### 3.2. Catalyst Preparation

Peel residues from potatoes bought from the local market were washed, chopped into small pieces, sieved, and dried at 70 °C for 24 h. Subsequently, they were pyrolyzed in a muffle furnace under different temperatures (i.e., 400, 600, and 800 °C) for 3 h in an N_2_ atmosphere to prevent combustion. Finally, the potato-derived biochars were labeled PPBC400, PPBC600, and PPBC800.

### 3.3. Characterization Methods

The morphology of the PPBC samples was explored using scanning electron microscopy (SEM) (JEOL 6300, Peabody, MA, USA) while their crystallographic structure was studied using X-ray diffraction (XRD) (Model, Company, Billerica, MA, USA) in a scanning range of 5 to 95 (2θ). PPBC samples were also characterized in terms of their specific surface area (SSA) utilizing Microtrac Belsorp Mini X equipment. Moisture and other substances adsorbed on the sample surfaces were removed in situ by applying vacuum under heating at 373 K for 1 h. Surface area measurements were determined using the standard B.E.T. equation within the nitrogen relative pressure range of 0.06 < P/P_0_ < 0.20. Fourier transform infrared (FTIR) experiments were performed employing a Nicolet 6700 apparatus (Thermo Fisher Scientific, Waltham, MA, USA), which was equipped with a diffuse reflectance (DRIFT) cell (Spectra Tech, Oak Ridge, TN, USA), an MCT detector, and a KBr beam splitter [70,71].

### 3.4. Experimental Procedure

Batch oxidation experiments were conducted at a room temperature of 25 ± 1 °C under atmospheric air pressure. In a typical procedure, 30 mg PPBC (500 mg/L) and 30 mg SPS (500 mg/L) were dispersed into a 60 mL solution (0.75 mg/L VEN) at inherent pH and the solution was shaken at 400 rpm to ensure a homogeneous reaction. The samples were collected at specific time intervals (0, 5, 10, 15, 30, 45, 60, 90, and 120 min), were rapidly quenched with methanol, and filtered through 0.22 μm PVDF filter before analysis. Control experiments with PPBC or persulfate alone were carried out under these conditions. All experiments were conducted in duplicate unless otherwise specified, with the variation between replicates remaining below 6%.

Using the optimum PPBC calcination temperature, the effects of PPBC dose (250–750 mg/L), SPS dose (250–750 mg/L), initial pH (3–9, adjusted either H_2_SO_4_ 1 M or NaOH 1 M), initial VEN concentration (0.75–1.5 mg/L), anions and humic acid ([HA] = 10.0 mg/L, [${HCO}_{3}^{-}$] = 250 mg/L, and [${Cl}^{-}$] = 250 mg/L), and water matrix (BW, WW) on the degradation of VEN were investigated. To investigate the reaction mechanism tert-butyl alcohol (t-BuOH), methanol (MeOH), and NaN_3_ were used at 9.54 mM.

For the experiments dedicated to the identification of transformation products (TPs), the following conditions were used: inherent pH, venlafaxine concentration of 5 mg/L, SPS concentration of 750 mg/L, and PPBC800 dosage of 500 mg/L.

### 3.5. Analytical Methods

VEN concentration was monitored using a Water Alliance 2695 HPLC instrument (Waters, Milford, MA, USA) equipped with a Kinetex C18 reversed phase column (2.6 μm particle size, 150 × 2.1 mm), and the UV wavelength was 226 nm. The column temperature was 45 °C, and the mobile phase combined 25% acetonitrile and 75% UPW with 0.1% phosphoric acid at a flow rate of 0.2 mL/min. The injection volume was 100 µL for each sample.

For the identification of potential transformation products (TPs), the samples were analyzed utilizing LC-ESI-TIMS-QTOF-MS. Analysis was carried out using ultrahigh-performance liquid chromatography (Elute LC series, Bruker Daltonics, Bremen, Germany) coupled to a hybrid trapped ion mobility-quadrupole time-of-flight system (timsTOF Pro 2, Bruker Daltonics, Bremen, Germany). Reversed-phase (RPLC) liquid chromatography was used for the separation and identification of TPs. In addition, hydrophilic interaction liquid chromatography (HILIC) was used for complementary identification of TPs. For screening of the candidate TPs of venlafaxine, suspect and non-target screening workflows were used. More details regarding the LC-HRMS system and data processing procedures are described in detail in the Supplementary Information (Section S1).

Radical and singlet-oxygen probes were analyzed by electron paramagnetic resonance EPR (JEOL X310, JEOL USA, Peabody, MA, USA) using an LC12 aqueous flat cell. Spectra were collected at 10 mW microwave power for hydroxyl and sulfate radicals and 1 mW for singlet oxygen with a 30 s scan time, employing 100 µL aliquots. 5,5-Dimethyl-1-pyrroline N-oxide (DMPO, CAS 3317-61-1) served as the spin-trapping agent for ^•^OH and ${SO}_{4}^{•-}$, while 2,2,6,6-tetramethylpiperidine (TEMP, CAS 768-66-1) was used to detect singlet oxygen.

### 3.6. Toxicity Evaluation of VEN and Its TPs-ECOSAR

The potential toxicity of venlafaxine and the detected transformation products towards aquatic organisms was evaluated using the Ecological Structure Activity Relationship (ECOSAR 2.2) software from the U.S. Environmental Protection Agency (US EPA). Employing a quantitative structure–activity relationship (QSAR) approach, ECOSAR establishes correlations between the structural characteristics of chemicals and their biological activity or toxicity. It is specifically designed to estimate acute and chronic toxicities for various aquatic organisms. Three different organisms were used to predict acute toxicity, with the determination of LC_50_ values (96 h for fish) and EC_50_ values (48-h for daphnia and 96-h for green algae). These representative species, originating from different environmental compartments and exhibiting varying sensitivities to pollutants, were selected to provide a more comprehensive assessment of the potential ecotoxicological impact of the treated effluent.

Additionally, chronic aquatic toxicity (ChV), which refers to the long-term adverse effects that a chemical substance can have on aquatic organisms over an extended duration of exposure, was considered in the overall assessment. The calculated toxicity values were classified according to the prescribed guidelines of the Globally Harmonized System of Classification and Labelling of Chemicals (GHS) [72]. Toxicity values falling within the range of 0 to 1.0 mg/L were assigned the category of “very toxic”, those ranging from 1 to 10 mg/L were classified as “toxic”, and between 10 and 100 mg/L were designated as “harmful”, whereas values exceeding 100 mg/L were categorized as “not classified for acute/long-term hazard”.

### 3.7. Life Cycle Analysis

#### 3.7.1. Goal and Scope Definition

The Life Cycle Assessment (LCA) of biochar production from potato peels was conducted in accordance with the principles and framework defined by ISO 14040 and ISO 14044 standards [73,74]. The goal of this study is to evaluate the environmental impacts associated with the thermal conversion of food processing waste (potato peels) into biochar, considering different pyrolysis temperatures (400 °C, 600 °C, and 800 °C). The analysis aims to provide insight into the energy requirements and corresponding environmental burdens of each thermal regime.

The system boundaries were set as “gate-to-gate”, focusing exclusively on the pyrolysis stage of biochar production. Upstream processes, such as potato cultivation and peel collection, were excluded, as the feedstock is considered a waste stream with no attributed environmental burdens (cut-off approach). This decision was made in order to isolate the contribution of energy input and processing temperature to the total impact. The functional unit (FU) was defined as 1 kg of biochar produced on a dry basis.

#### 3.7.2. Life Cycle Inventory (LCI)

The life cycle inventory was developed based on experimental data obtained from pilot-scale pyrolysis trials using raw potato peels with 40% moisture content. For each temperature condition (400 °C, 600 °C, and 800 °C), the biochar yield was measured (35%, 35%, and 28%, respectively), and the corresponding thermal energy input was calculated considering the latent heat of moisture removal, specific heat capacity of biomass, and temperature difference. Two different energy supply systems were modeled for natural gas combustion and electricity from European grid mix.

The specific energy requirements (in MJ/kg biochar) were calculated for each scenario and converted into environmental burdens using characterization factors provided by the GaBi LCA software database. The inventory data also included the emission factors for each energy type, as reported in the Gabi professional database (version January 2016).

#### 3.7.3. Life Cycle Impact Assessment (LCIA)

The environmental assessment was performed using the GaBi LCA software, employing the CML2001 impact assessment methodology (version January 2016). The selected impact categories for this study were Global Warming Potential (GWP) over a 100-year timeframe (kg CO_2_-eq) and Abiotic Depletion Potential–fossil fuels (ADP fossil) (MJ). These two indicators were chosen as the most relevant to the environmental performance of energy-intensive thermochemical processes and are widely used in comparable LCA studies. Other indicators such as acidification, eutrophication, and toxicity potential were not included, as the focus of this assessment was limited to energy use and climate-related burdens [75].

## 4. Conclusions

In this work, a biochar produced from potato peels was synthesized at different temperatures, characterized, and examined for the persulfate-assisted degradation of the pharmaceutical VEN. According to the results, although the biochar produced at 800 °C exhibited negligible surface area, it demonstrated higher efficiency in VEN degradation, despite its low surface area, likely due to the synergy of oxygenated surface functionalities and more developed conjugated/graphitic domains that facilitate surface-mediated electron transfer.

As expected, the degradation of VEN was enhanced with increased persulfate and biochar loading. The efficiency of the system was promoted under acidic conditions; however, the proposed process demonstrated high efficiency across a wide pH range. Interestingly, only a slight retardation was observed in the presence of both inorganic ions and organic matter (simulated as humic acids), while significant activity was also observed in the experiment performed in secondary effluent. These findings suggest that the system operates effectively under neutral to mildly alkaline conditions, which are most relevant for practical applications in water and wastewater treatment.

Scavenging tests, together with EPR, indicate a mixed mechanism dominated by non-radical pathways, with radicals contributions being minor. The analysis of the samples by LC-TIMS-QToF-MS enabled the identification of five different transformation products. Most of these transformation products have been structurally elucidated, and potential reaction pathways have been proposed. Among them, N-desmethyl venlafaxine was the most abundant TP, exhibiting a continuous and steady increase in its abundance over time.

Utilizing the ECOSAR software (version 2.2) to estimate the toxicity of venlafaxine and its identified TPs gave interesting results, indicating that all the detected TPs showed lower toxicity than the parent compound. Furthermore, acute toxicity values for most of the TPs and the chronic toxicity values of VEN_TP291 were classified as “harmful” towards all the tested organisms. However, further research is needed to thoroughly evaluate and understand the environmental risks associated with VEN and its degradation products.

Finally, according to the LCA results regarding ADP-fossil and GWP indicators, natural gas systems consistently present lower ADP values compared to electricity-based systems. In addition, the biochar produced at 800 °C, which demonstrated the highest efficiency, also exhibited the largest environmental footprint, indicating the need for future research toward the development of strategies that will provide an integrated assessment of materials, technologies, and systems based on both efficiency and environmental footprint.

## Figures and Tables

**Table 1:** Primary crystallite size of PPBC samples.

Sample	Primary Crystallite Size (Å)
PPBC 400	41.6
PPBC 600	41.2
PPBC 800	36.3

For the calculation of the primary crystallite size, the analysis was performed on the most intense diffraction peak, located at approximately 28.1°.

**Table 2:** k_app_ values for 0.75 mg/L VEN adsorption and degradation for the examined biochars. Experimental conditions [PPBC] = 500 mg/L and [SPS] = 500 mg/L in UPW. In all cases, R^2^ > 0.97.

Sample	$k_{adsorption}$, min^−1^	$k_{oxidation}$, min^−1^
PPBC 400	0.0042	0.0029
PPBC 600	0.0003	0.0012
PPBC 800	0.0017	0.0389

**Table 3:** k_app_ values for 0.75 mg/L VEN adsorption and degradation for the examined pH solutions. Experimental conditions [PPBC800] = 500 mg/L and [SPS] = 500 mg/L in UPW. In all cases, R^2^ > 0.99.

pH	$k_{adsorption}$, min^−1^	$k_{oxidation}$, min^−1^
3	0.0032	0.1136
Inherent pH (7)	0.0017	0.0389
9	0.0028	0.0352

**Table 4:** k_app_ values for 0.75 mg/L VEN degradation for several scavengers at inherent pH. Experimental conditions are [PPBC800] = 500 mg/L, [scavenger] = 9.54 mM, and [SPS] = 500 mg/L in UPW, Molar ratio $\frac{\left[scavenger\right]}{\left[SPS\right]}=4.54$.

pH	$k_{app}$, min^−1^	R^2^
Without scavenger	0.0389	0.99
MeOH	0.0058	0.98
t-BuOH	0.0063	0.98
NaN_3_	0.0030	0.99

**Table 5:** LC-HRMS data for venlafaxine and its transformation products.

Compound	Ion Mode	Retention Time (min)	Principal Ion-Molecular Formula	Principal Ion- *m*/*z*	Err (ppm)	Fragments Ions- Molecular Formula	Fragments Ions- *m*/*z*	Err (ppm)
**Venlafaxine (VEN)**	ESI+	5.47	C_17_H_28_NO_2_^+^	278.2124	−1.2	C_8_H_9_O^+^	121.0648	−3.8
						C_10_H_11_O^+^	147.0804	−2.8
						C_11_H_11_O^+^	159.0804	−2.8
						C_12_H_13_O^+^	173.0961	−1.0
						C_15_H_19_O^+^	215.143	0.0
						C_17_H_26_NO^+^	260.2009	−0.6
**VEN_TP263a**	ESI+	4.51	C_16_H_26_NO_2_^+^	264.196	−2.1	C_9_H_11_N^+^	133.0886	20.1
						C_8_H_11_^+^	107.0855	4.0
						C_8_H_11_O^+^	123.0804	1.5
						C_6_H_9_O^+^	97.0648	5.2
						C_6_H_9_O^+^	201.1274	6.9
**VEN_TP263b**	ESI+	5.53	C_16_H_26_NO_2_^+^	264.1958	−2.3	C_8_H_9_O^+^	121.0648	0.3
						C_10_H_11_O^+^	147.0804	1.3
						C_11_H_11_O^+^	159.0804	1.8
						C_15_H_19_O^+^	215.143	1
**VEN_TP293**	ESI+	6.27	C_17_H_28_NO_3_^+^	294.2063	−3.7	C_8_H_9_O^+^	121.0648	−2.3
						C_15_H_19_O^+^	215.1430	−1.8
						C_6_H_9_^+^	81.0699	0.7
						C_10_H_7_NO^+^	157.0522	14.8
						C_11_H_16_NO^+^	178.1226	−1.8
**VEN_TP291**	ESI+	7.85	C_17_H_26_NO_3_^+^	292.1912	1.0	C_8_H_9_O^+^	121.0648	1.2
						C_15_H_19_O^+^	215.1430	−0.7
						C_14_H_19_O_2_^+^	219.1380	0.4
						C_11_H_11_O^+^	159.0804	−3.1
**VEN_TP275**	ESI+	4.73	C_17_H_26_NO_2_^+^	276.1958	−2.0	C_10_H_12_NO^+^	162.0913	−0.8
						C_9_H_9_NO^+^	147.0679	1.6
						C_11_H_14_NO^+^	176.107	−0.9

**Table 6:** Aquatic toxicity assessment of venlafaxine and its transformation products.

	Acute Toxicity			Chronic Toxicity		
Compound	Fish	Daphnia	Green Algae	Fish	Daphnia	Green Algae
	LC_50_–96 h	LC_50_–48 h	EC_50_–96 h	ChV	ChV	ChV
	mg/L					
**Venlafaxine**	16.1	10.3	12.5	1.81	1.40	4.27
**VEN_TP263a**	48.9	29.7	29.1	5.17	3.48	8.83
**VEN_TP263b**	23.7	14.9	16.6	2.60	1.91	5.43
**VEN_TP293**	96.8	57.3	50.5	9.93	6.26	14.5
**VEN_TP291**	1.17 × 10^3^	621	345	10.0	49.7	77.1
**VEN_TP275**	20.6	13.0	15.1	2.28	1.17	5.01
Harmful	10–100 mg L^−1^					

## Data Availability

Dataset available on request from the authors.

## Supplementary Materials

The following supporting information can be downloaded at https://www.mdpi.com/article/10.3390/molecules30183720/s1. Figure S1. Experimental MS/MS spectra of [M + H]+ ions of VEN and its detected TPs acquired by data-dependent acquisition mode. Table S1. Advanced oxidation processes for venlafaxine (VEN) removal.
