# Supplementary material for: Effective Degradation of Venlafaxine via Biochar Activated Persulfate: Kinetics, Transformation Products, and Toxicity Assessment
Source: Molecules. 2025 Sep 12;30(18):3720. doi: 10.3390/molecules30183720 (PMC12472649; doi:10.3390/molecules30183720)
Supplement: Supplementary file 1 [file molecules-30-03720-s001.zip › molecules-3785381-supplementary.pdf]

Supplementary Information

Effective Degradation of Venlafaxine via Biochar Activated Persulfate: Kinetics, Transformation Products, and Toxicity Assessment

Alexandra A. Ioannidi [1], Eleni I. Panagopoulou [2], Konstantinos Kouvelis [1], Dimitrios Ladakis [3], Athanasia Petala [4], Marilena E. Dasenaki [5], Nikolaos S. Thomaidis [2], Dionissios Mantzavinos [1], Zacharias Frontistis [6,*] and Olga S. Arvaniti [3,*]

1 Department of Chemical Engineering, University of Patras, Caratheodory 1, University Campus, GR-26504 Patras, Greece; alex.ioannidi@chemeng.upatras.gr (A.A.I.); koskouv@chemeng.upatras.gr (K.K.), mantzavinos@chemeng.upatras.gr (D.M.)

2 Department of Chemistry, Laboratory of Analytical Chemistry, National and Kapodistrian University of Athens, Panepistimioupolis Zografou, GR 15771, Athens, Greece; elenapanag@chem.uoa.gr (E.I.P.); ntho@chem.uoa.gr (N.S.T.)

3 Department of Agricultural Development, Agrofood and Management of Natural Resources, National and Kapodistrian University of Athens, Psachna 34400, Greece; dladakis@agro.uoa.gr (D.L.)

4 Department of Environment, Ionian University, GR-29100 Zakynthos, Greece; apetala@ionio.gr

5 Department of Chemistry, Laboratory of Food Chemistry, National and Kapodistrian University of Athens, Panepistimiopolis Zographou, 15771 Athens, Greece; mdasenaki@chem.uoa.gr

6 Department of Chemical Engineering, University of Western Macedonia, GR-50132 Kozani, Greece

* Correspondence: zfrontistis@uowm.gr (Z.F.); oarvaniti@agro.uoa.gr (O.S.A.)

Section S1: Details regarding instrumental analysis and data processing procedures for the identification of transformation products

Number of Figures: 1

Number of Tables: 1

Section S1: Details regarding instrumental analysis and data processing procedures for the identification of transformation products

1.1 Analytical procedures

Ultra-high-performance liquid chromatography (Elute LC series, Bruker Daltonics, Bremen, Germany) coupled to a hybrid trapped ion mobility-quadrupole time-of-flight system (timsTOF Pro 2, Bruker Daltonics, Bremen, Germany) was used. Chromatographic separation was performed on an Elute UHPLC equipped with η Intensity Solo 1.8 C18-2 100 x 2.1 column (Bruker, Germany), thermostated at 40 °C. In positive ionization mode, the mobile phases consisted of water, methanol (99:1 v/v, solvent A) and methanol (solvent B), both containing 5 mM ammonium formate and 0.01% formic acid. In negative ionization mode, the mobile phases consisted of water methanol (99:1 v/v, solvent A) and methanol (solvent B), both containing 5 mM of ammonium acetate. A gradient elution program was applied in both ionization modes (Table 3), starting with 4% B (flow rate of 0.2 mL min^{-1}) for 0.1 min, which was increased to 18.3% for 0.9 min and then to 50% (flow rate of 0.223 mL min-1) for another 1.5 min. After that, B was increased to 99.9% (flow rate of 0.4 mL min^{-1}) for another 12.5 min. Then, B was kept constant for 2 min (flow rate of 0.48 mL min^{-1}); initial chromatographic conditions were restored through the re-equilibration of the column for 3 min. The injection volume was set to 1 μL in positive ionization mode and 3 μL in negative ionization mode.

The TIMS-QTOF-MS system was equipped with an electrospray ionization interface (ESI), operating in positive and negative modes. The ionization parameters were as follows: capillary voltage 2500 V in positive and negative mode; nebulizer gas (N_2) pressure 2 bar; drying gas flow 10 L min^{-1}; endplate offset 500 V and dry temperature 220 °C.

The external mass and mobility calibration was performed with the manufacturer's solution, sodium formate 10 mM: Agilent ESI-L Low Concentration Tuning Mix (1:3, v/v). The QTOF external calibration was performed using sodium formate 10 mM daily in water/isopropanol, which included cluster ions with formulas Na(NaCOOH)1−18 in the m/z range of 20–1300. TIMS ion charge control was set to 7.5e6. For mobility calibration, Agilent ESI-L Low Concentration Tuning Mix was used (Agilent Technologies, USA). In the positive mode, the mobility calibration ranges between 0.5446 and 0.9915 V·s/cm^2, while in the negative mode it ranges between 0.6690 and 0.8824 V·s/cm^2.

The samples were analyzed utilizing both data-independent acquisition mode (DIA, bbCID) and data-dependent acquisition mode (DDA, PASEF). The TIMS-QTOF-MS system was operated in PASEF acquisition mode with a mass scan range of 20-1300 m/z and $1/k_0$ range of 0.40-1.37 Vs cm^{-2}. Regarding TIMS, a duty cycle of 100% can be achieved thanks to a dual TIMS setup, given that accumulation and ramp times are kept stable. For the current method, accumulation and ramp time were set at 100 msec each, while a total cycle of 0.53 sec was also selected, resulting in one full TIMS-MS scan and two PASEF MS/MS scans.

The samples were also analyzed with an ultra-high-performance liquid chromatography system with an HPG-3400 pump (UHPLC, Dionex UltiMate 3000 RSLC, Thermo Fisher Scientific). In tandem, a QToF mass spectrometer (Maxis Impact, Bruker Daltonics, Bremen, Germany) was used for the analysis of the samples. The QToF system was equipped with an electrospray ionization interface (ESI), operating

in positive and negative ionization modes. Hydrophilic interaction liquid chromatography (HILIC) was used as a complementary method for the orthogonal identification of TPs for the separation and identification of TPs. In HILIC, chromatographic separation was performed on a Waters ACQUTIY UPLC BEH Amide column (2.1 x 100 mm, 1.7 μm). A guard pre-column of the same packaging material was used, and the column was thermostated at 40 °C. For positive ionization mode, the mobile phases were water (solvent A) and ACN: H_2O, 95:5 (solvent B), both amended with 1 mM ammonium formate and 0.01% formic acid. For negative ionization mode, the mobile phases consisted of water (solvent A) and ACN: H_2O, 95:5 (solvent B) both acidified with 10 mM ammonium formate. The adopted gradient elution program, for both ionization modes, started with 100% B for 2 min, decreasing to 5% for 10 min, and kept constant for the following 5 min. The initial conditions were restored within 0.1 min and allowed to re-equilibrate for 8 min. The flow rate was 0.2 mL min^{-1}. The injection volume was set to 5 μL. The QToF system was equipped with an electrospray ionization interface (ESI), operating in positive and negative modes. The operation parameters of ESI were the following: capillary voltage, 2500 V for positive and 3000 V for negative mode; endplate offset, 500 V; nebulizer pressure, 2 bar (N_2); drying gas, 8 L min^{-1} (N_2); and drying temperature, 200 °C. Both data-dependent (AutoMS) and data-independent (broadband collision-induced dissociation (bbCID)) MS/MS spectra were recorded.

1.2 Data processing for the identification of TPs

Both suspect and non-target screening approaches were followed for the identification of the potential transformation products. However, some of the information, such as their formula, the structure, and the degradation reaction, was available in previously reported studies or could be provided by metabolite prediction tools. For the suspect screening, an in-house-built database of the candidate metabolites was created. Database compilation was achieved utilizing the in silico tool Metabolite Predict (Metabolite Tools 2.0, Bruker Daltonics, Bremen, Germany). Additional information for some TPs obtained from the already published literature or common reactions was included in the database. Apart from Metabolite Predict, BioTransformer was applied in this study, which is included in MetaboScape software (MetaboScape 2023, Bruker Daltonics, Bremen, Germany). This tool is specifically designed for predicting the metabolism of small molecules with known structures. It annotates the anticipated transformation products in the feature table of MetaboScape based on m/z and isotopic profile, providing a suspect list of potential TPs. Notably, after the annotation via BioTransformer, the features with available MS/MS spectra were automatically processed using the in silico fragmentation tool MetFrag. MetFrag was used in both suspect and non-target screening workflows to support the annotation.

The following step was the screening of all the samples in positive and negative modes, in both RPLC and HILIC, with the aforementioned database with the suspect compounds using Data Analysis 6.0 and TASQ Client 2022 and Metaboscape 2022 software (Bruker Daltonics, Bremen, Germany). The identification of the TPs through suspect screening relied on criteria such as a threshold in peak area and ion intensity, a mass accuracy threshold, and a good isotopic pattern fitting. The absence (or presence at very low level) of a peak with a similar retention time RT (± 0.2min) from

the control was also examined. Peaks that were present in the control samples were disregarded from further processing. The introduced filtering criteria aim to reduce false-positive identifications, while, at the same time, minimizing the false-negative results. A non-target screening approach was also followed for the investigation of additional TPs in the samples, which were not included in the suspect database, and the samples were screened for additional TPs. The treated samples at different time points were compared with the sample at the beginning of the experiment (zero-time sample). Background subtraction was carried out using Data Analysis software and the "expose" algorithm, which accounts for tolerances in retention time (±0.2 min) and mass accuracy (± 0.005 m/z), as well as for intensity ratios (value > 5) between the treated samples and the zero-time sample. Relevant peaks that were neither presented in the zero-time sample nor in the existing suspect database were selected from the generated peak list. In the non-target approach, the main distinction was the lack of a priori knowledge regarding the molecular formula of these compounds. To determine plausible molecular formulas, the Smart Formula tool from Bruker Daltonics (Data Analysis, Bruker Daltonics) was utilized. The assignment of molecular formulas to the unknown peaks considered factors such as mass accuracy, isotopic pattern, element restrictions (C, H, N, O, and S), hydrogen to carbon ratio (H/C) ranging from 0 to 3, presence of rings and double bonds, and electron configuration. Furthermore, the data processing was also performed using MetaboScape software (MetaboScape 2023, Bruker Daltonics, Bremen, Germany), compatible with both LC-QTOF and LC-TIMS-QTOF data. Data processing from LC-QTOF analysis was performed with the algorithm considering 3 particular parameters through the peak-picking step, namely retention time, mass-to-charge ratio (m/z), and intensity. For LC-TIMS-QTOF data, the Time-aligned Region complete Extraction ("T-ReX 4D") processing workflow of MetaboScape was employed, incorporating the calculation of CCS alongside the aforementioned parameters during peak-picking. The detection of a tentative TP in both chromatographic systems (RPLC and HILIC) and ESI polarities was also examined. This step was not mandatory for the tentative identification; however, it may provide additional experimental evidence.

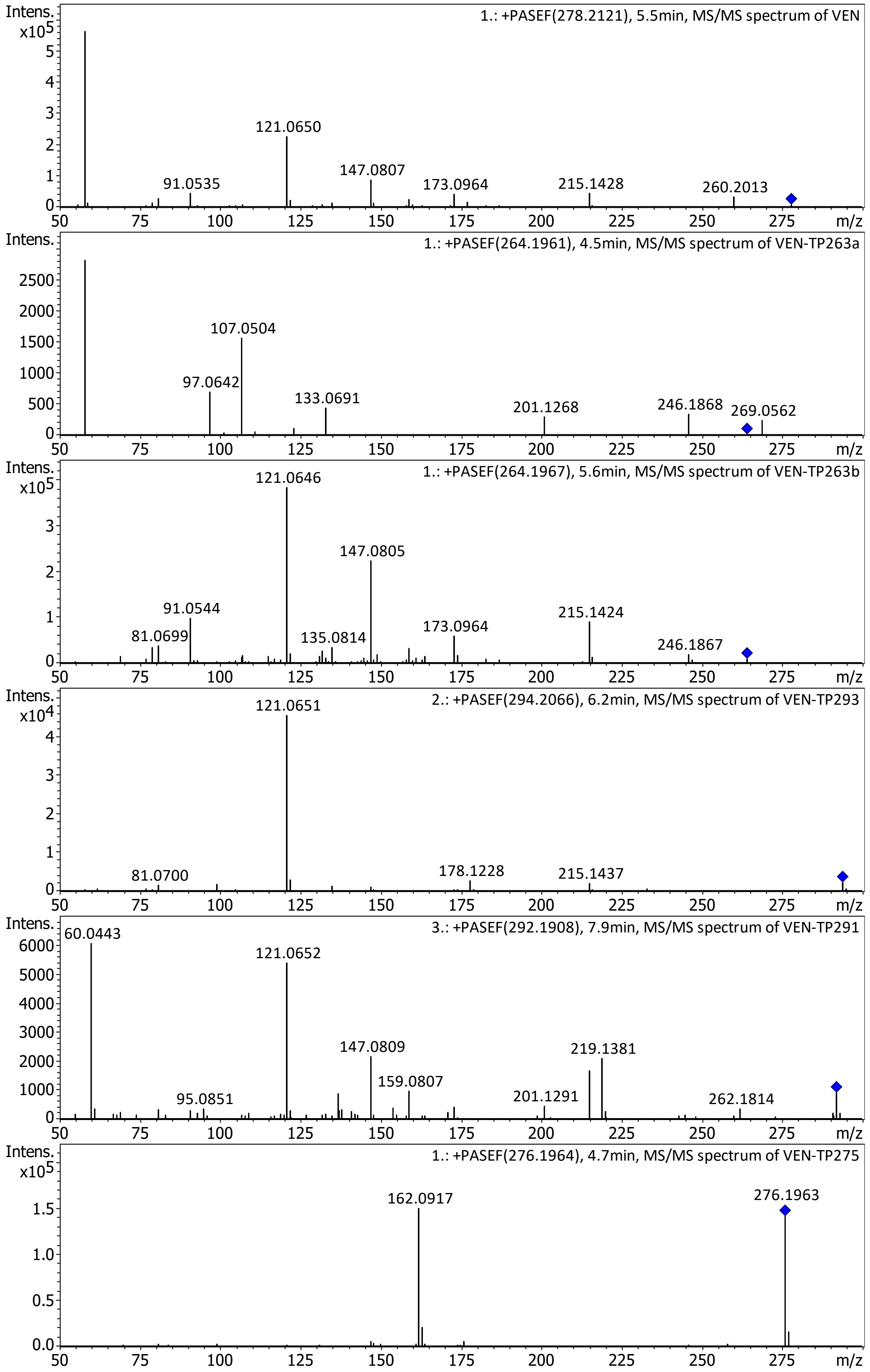

Figure S1. Experimental MS/MS spectra of $[M+H]^+$ ions of VEN and its detected TPs acquired in data-dependent acquisition mode.

Table S1 Advanced oxidation processes for venlafaxine (VEN) removal.

Process	Conditions	Removal	Ref
UV-C/H_2O_2	[VEN]=20 mg/L, [H_2O_2]=700 mg/L, 33 J cm^{-2}	99.9 % at 5 min	31
UV-C/ Chlorine	[VEN]=2 mg/L, [Chlorine]=6 mg/L, 75 W low pressure mercury UV lamps (Irradiation: 4.93 × 10^{-5} Einstein/sec)	76.1% after 30 min (UV-C/Chlorine) and 39.6% after 30 min (Chlorine)	32
UV-A /TiO_2	[VEN]=2.5 mg/L, 9W UV-A lamp, (Irradiation: 1.18 × 10−4 einstein min^{-1}), [TiO_2]=400 mg/L, pH =4	100% After 20 min	33
Solar Photo Fenton	[VEN]=100 20 mg/L, pH=3, [Fe^{2+}]=20 mg/L, [H_2O_2]=50 mg/L, optimal Fe/H_2O_2=0.4, Xenon lamp 900 Watt/m^2	90% at 10 min	34
Solar Photolysis	[VEN]=100 mg/l, Xenon lamp 900 Watt/m^2	12% after 24 hours	34
Electro peroxone	[VEN]=20 mg/L, [O_3]$_{gas}$=20 mg/L, pH= 7.9, gas flow 250 L/min, current 200 mA	100 at 15 min	35
Fe^{2+}/Cys/PS	[PS] =15 mM, [Fe^{2+}] = 1 mM, [Cys] = 0.25 mM, [Ven] = 10 mg/L, pH =3	100% at 30 min	36
PTFE-supported rGO membranes/PS	Continuous mode (24 h), [VEN]= 250 µg/L.,[rGO]= 15 mg, [PS] =1 mM, flow rate = 0.1 mL/min, pH = 7.0	94% between 2 and 24 hours	37
CNT@Ni+Fe/Al_2O_3-cp-PVDF membranes/persulfate	CNT@Ni+Fe/Al_2O_3-cp-PVDF membranes , Continuous mode, [VEN]= 100 µg L1, [PS] = 250 mg/L, pH = 7 , Q = 0.1 mL/min, surface water	95% at 90 min	38
Potato peel residual biochar/persulfate	[VEN]= 750 µg/L, PS] = 250 mg/L, [BC]=500mg/L, pH = 7	100% at 120 min	This study
